# Motivational strategies to improve adherence to dementia preventive lifestyle changes

**DOI:** 10.1002/alz70860_102949

**Published:** 2025-12-23

**Authors:** Esther Brill, Giovanni B. Frisoni, Dag Aarsland, Stefan Klöppel

**Affiliations:** ^1^ University Hospital of Old Age Psychiatry and Psychotherapy, Bern, Bern, Switzerland; ^2^ Geneva Memory Center, Department of Rehabilitation and Geriatrics, Geneva University Hospitals, Geneva, Geneva, Switzerland; ^3^ Department of Old Age Psychiatry, Kings College, London, London, United Kingdom

## Abstract

**Background:**

Dementia represents a significant global health challenge, necessitating innovative prevention strategies. Lifestyles aimed at reducing dementia risk typically combine physical and cognitive training, nutritional adaptations, and social engagement. However, sustainable lifestyle changes require robust motivation and volition. Acting in accordance with one's values enhances motivation, as values are intrinsically rewarding and create stable behavior‐outcome relationships. Despite the efficacy of well‐structured interventions like FINGER, long‐term adherence remains a challenge, with fewer than half of participants completing all intervention components.

**Method:**

We developed a value‐based motivational framework integrated with digital tools, including smartphone‐based reminders, ecological momentary assessments, and just‐in‐time adaptive interventions (JITAIs). Further, we assessed feasibility of digital tools in older adults with subjective or mild cognitive decline in a feasibility study (*n* = 15). Our framework is designed to align individual preferences and values with lifestyle goals, employing motivational interviewing and SMART goal‐setting techniques. We previously implemented this approach as a manualized consultation in the memory clinic and now evaluate the effects of smartphone‐delivered personalized, value‐based communication styles compared to neutrally framed statements on the intended adherence to dementia preventive behaviors.

**Result:**

The feasibility pilot demonstrates that smartphone‐based JITAIs are feasible, well adhered to and generally well‐accepted by the target population. Preliminary findings of the in‐clinic consultations indicate the potential of value‐based motivational strategies to improve intended adherence to lifestyle changes. The quantitative assessment of the impact of positively framed value‐based motivational strategies on participants' adherence to a dementia‐preventive lifestyle is currently ongoing.

**Conclusion:**

Value‐based motivational strategies, supported by digital tools and positively‐framed communication, hold high potential for fostering adherence to dementia‐preventive lifestyle changes. These approaches are clinically relevant and resource‐efficient, making them suitable for integration into large‐scale prevention programs. By leveraging motivation and volition, this framework addresses key barriers to long‐term behavior change and contributes to healthy aging. The anticipated findings will provide valuable insights into effective strategies for promoting healthy aging and may inform future public health interventions aimed at this vulnerable population.

